# Clinical-epidemiological characteristics of deaths due to dengue during an outbreak in northern Peru

**DOI:** 10.17843/rpmesp.2023.401.12148

**Published:** 2023-03-31

**Authors:** Néstor Luque, Catia Cilloniz, María J. Pons, Fernando Donaires, Roger Albornoz, Moisés Mendocilla-Risco, Manuel Espinoza

**Affiliations:** 1 School of Human Medicine, Faculty of Health Sciences, Universidad Peruana Unión (UPeU), Lima, Peru. Universidad Peruana Unión School of Human Medicine Faculty of Health Sciences Universidad Peruana Unión (UPeU) Lima Peru; 2 Universidad Continental, Lima, Peru. Universidad Continental Lima Peru; 3 Re-emerging infectious diseases group, Universidad Científica del Sur, Lima, Peru. Universidad Científica del Sur Re-emerging infectious diseases group Universidad Científica del Sur Lima Peru; 4 Instituto Nacional de Salud, Lima, Peru. Instituto Nacional de Salud Lima Peru; 5 Universidad Nacional Mayor San Marcos, Lima, Peru. Universidad Nacional Mayor de San Marcos Universidad Nacional Mayor San Marcos Lima Peru

**Keywords:** Dengue fever, Mortality, Medical Records, Epidemiology, Peru

## Abstract

Objective: To describe the clinical-epidemiological characteristics of patients who died from dengue during 2017. Methods: We conducted a retrospective study of the information related to cases of dengue deaths in the department of Piura. Results: We reviewed 24 medical records. Sixty-seven percent were women and 3 (12.5%) were pregnant. Diabetes (12.5%) and hypertension (16.7%) were the most frequent comorbidities. Previous dengue fever was reported in only 12.5%. The time from health care and death was 4.10 ± 5.34 days. Red blood cell transfusions were performed in 45.8%, plasma in 25%, platelets in 16.8% and cryoprecipitate in 16.8% of cases. Crystalloid therapy (91.7%) and treatment with vasoactive drugs (70.8%) were also frequent. In conclusion, mortality from severe dengue fever was predominantly in adult women, and the time of care from the first health facility to a specialized unit was prolonged.

## INTRODUCTION

In the Americas, more than 2.7 million cases of dengue were reported during 2019, of which 22,127 were severe cases, with 1206 deaths ^1^. The number of deaths from dengue increased during the last decade; several countries reported high rates of fatal cases during recent epidemics ^2^. Dengue has been a growing health problem since its re-entry into Peru in 1990, with reports showing the circulation of all four serotypes ^3^. In Peru, during 2017, epidemiological reports showed 68,290 confirmed and probable cases, of which 27,249 (35.8%) were confirmed by laboratory tests, 79 of the confirmed cases died, and of these 43 (54.4%) were from the Piura region ^4^.

In Peru, the 2017 Coastal El Niño was quite destructive, particularly for the northern region during almost three months ^5^. According to the World Health Organization, it is essential to have global epidemiological data to predict and identify severe cases of dengue ^6^. For this reason, this study aimed to describe the clinical and epidemiological characteristics of patients who died from dengue in healthcare facilities in the Piura region, northern Peru, in 2017.

**KEY MESSAGES**

Motivation for the study. To describe the characteristics of patients who died from severe dengue fever during the 2017 El Niño in Piura.

Main findings. Mortality from severe dengue was higher in adult women. First contact with healthcare took place mostly in higher level hospitals. Admission to a specialized unit was late for severe dengue cases.

Implications. Control of dengue fever involves several aspects, such as, access to health, prevention, water availability, vector control and education; therefore, it is important to strengthen public health policies in this regard. In order to achieve this goal, local and central government sectors must be involved.

## THE STUDY

### Population and study design

A retrospective study was conducted to review the medical records of patients who died of dengue in the Piura region up to epidemiological week 42 of 2017, which includes a dengue outbreak. The Epidemiology Area of the Regional Health Directorate of Piura (DIRESA-Piura), provided 32 medical records, eight of which were excluded (duplicate records, no medical death certificate, illegible information and incomplete information), therefore 24 were finally included.

### Dengue case definition

Dengue was diagnosed according to the clinical and epidemiological criteria established by the technical guideline of the Peruvian Ministry of Health (MINSA) ^7^. All cases had positive results in one or more of the diagnostic tests, either the enzyme-linked immunosorbent assay (ELISA) for the nonstructural glycoprotein NS1, or the detection of IgM or IgG antibodies with the Peruvian TARIKI-DENGUE kit and Standard Diagnostics BIOLINE Dengue Duo, respectively.

### Data collection

All cases were from the Piura region. The physicians in charge filled the MINSA forms and the registry. A data collection instrument was used to obtain information from the medical records. We obtained epidemiological data such as age, sex, pathological history, history of dengue, time of illness, place of care, characteristics of the time of care between admissions to healthcare facilities; signs and symptoms such as fever (axillary temperature higher than 38°C), headache, retroorbital pain, myalgia, arthralgia, malaise, others; vital functions; fluid management; life and hematological support; laboratory tests (automated equipment was used to assess blood gases) and use of antipyretic drugs and antibiotics.

### Statistical analysis

Continuous variables were reported with mean and standard deviation (SD) or median and interquartile range (IQR), depending on their normal distribution. Categorical variables were reported with frequencies and percentages. All statistical analyses were performed with the SPSS 21 statistical program.

### Ethical aspects

This study was approved by the Research Ethics Committee of the Universidad Peruana Unión (UPeU) (code: 2022-CEUPeU-001).

## FINDINGS

### Diagnosis and care of dengue cases

Of the 24 cases, 62.5%, 70.8% and 41.7% were positive by NS1 (ELISA), IgM (ELISA) and IgG (ELISA), respectively. According to medical records, 8 (33.3%) deaths occurred at MINSA’s Santa Rosa II-2 Hospital, 6 (25.0%) at EsSalud’s Cayetano Heredia III Hospital, 5 (20.8%) at other EsSalud hospitals, 1 (4.2%) at the Armed Forces health facility, and 4 (16.7%) occurred at a primary care center.

### Clinical and epidemiological characteristics of dengue deaths

The epidemiological characteristics of the patients who died from dengue are described in Table 1. Mortality in female patients was higher; 3 (12.5%) of the deceased were pregnant. Comorbidities such as diabetes mellitus and arterial hypertension were reported in 12.5% and 16.7%, respectively; corticosteroids were not used prior to admission in any case. Four participants (16.7%) were identified as having possible previous contact with a patient with dengue fever (1 case with febrile syndrome and 3 cases with a previous history of dengue fever). According to the clinical diagnoses at admission, 6 (25%) had dengue with warning signs and 10 (42%) had severe dengue. The mean time elapsed between the first admission to a healthcare facility and death was 4.10 (SD 5.34) days (Table 1).

**Table 1:** Epidemiological characteristics of cases of death due to dengue.

Variables	n (%)
Age (years) ^a^	46 (25.0) (13-88) ^b^
Sex	
Female ^c^	16 (67.0)
Male	8 (33.0)
Comorbidities	
Diabetes *mellitus*	3 (12.5)
Arterial hypertension	4(16.7)
Others	6(25.0)
No information	11(45.8)
Previous history of dengue fever	
Yes	3 (12.5)
Fever syndrome	1 (4.2)
No information	20 (83.3)
Diagnosis at admission (first HF)	
Dengue with alarm symptoms	6 (25.0)
Severe dengue	10 (41.6)
Fever syndrome	4 (16.7)
Other diagnosis ^d^	4 (16.7)
Time of care (days)	
From HF to death ^a^	4.10 (5.34)
From onset of illness to death ^a^	7.93 (5.97)

a Mean and standard deviation, ^b^ minimum-maximum, ^c^ three women were pregnant, ^d^ severe community-acquired pneumonia (2 cases) and pregnant women with severe preeclampsia (2 cases).

HF: healthcare facility.

The clinical characteristics on admission are described in Table 2. The most frequent clinical manifestations were related to the febrile phase of dengue fever: fever (87.5%) and systemic symptoms such as headache (33.3%), nausea/vomiting (58.3%), abdominal pain (45.8%), arthralgia (33.3%) among others. In addition, vital signs on admission revealed tachycardia (mean heart rate of 108 beats per min), and polypnea (mean respiratory rate of 25).

**Table 2:** Clinical characteristics at admission related to deaths due to dengue.

Variables	n (%)
Signs and symptoms	
Fever	21 (87.5)
Headache	8 (33.3)
Retroorbital pain	3 (12.5)
Myalgia	1 (4.2)
Arthralgia	8 (33.3)
General malaise	9 (37.5)
Abdominal pain	11 (45.8)
Rash	1 (4.2)
Nausea/vomiting	14 (58.3)
Petechiae	2 (8.3)
Jaundice	2 (8.3)
Hematemesis	1 (4.2)
Diarrhea	5 (20.8)
Convulsions	3 (12.5)
Respiratory insufficiency	2 (8.3)
Black stools	2 (8.3)
Consciousness alterations	3 (12.5)
Other ^a^	4 (16.7)
Vital signs (on admission)	
Body temperature (°C) ^b^	37.8 (0.9)
Heart rate (pulse/min) ^b^	108 (19.5)
Mean arterial pressure (mmHg) ^b^	75 (21.7)
Respiratory frequency (min) ^b^	25 (10.3)
Glasgow Scale ^b^	13 (2.9)

a Ascites (one case), pallor (one case), profuse sweating (one case) and pleural effusion (one case), ^b^ mean and standard deviation.

Laboratory results are described in Table 3. The hemogram was performed within 48 hours of admission and showed wide variability, this may have been due to the fact that the data from peripheral healthcare facilities were obtained manually and data from greater complexity facilities were obtained using systematized methods. The results showed a decreasing trend of the platelet count. Lactate levels were found to be high. Results showed that transaminase enzymes (AST and ALT) were high, suggesting hepatic alterations; INR levels were also high. Half of the patients had thrombocytopenia.

**Table 3:** Results of laboratory parameters of deaths due to dengue.

Variables	Median (IQR)
Hematological parameters	
Leucocytes/mm^3^ (n=24)	6620 (4142-11692)
Hematocrit (%) (n=24)	41.0 (29.7-45.5)
Hemoglobin (gr/dL) (n=24)	13.6 (10.3-15.3)
Creatinine (mg/dL) (n=21)	1.3 (0.9-2.2)
Urea (mg/dL) (n=22)	53.5 (29.5-71.5)
Serum sodium (meq/L) (n=19)	140.3 (132-142)
Serum potassium (meq/L) (n=19)	4.2 (3.85-5.45)
Total bilirubin (mg/dL) (n=18)	0.7 (0.26-3.59)
Direct bilirubin (mg/dL) (n=18)	0.4 (0.16-2.48)
Indirect bilirubin (mg/dL) (n=18)	0.3 (0.12-0.79)
Platelets /mm^3^ (n=23)	105,000 (47,000-136,500)
AST (mg/dL) (n=18)	504.0 (87.2-1694)
ALT (mg/dL) (n=18)	432.0 (82.2-1343)
Prothrombin time (seg) (n=11)	21.5 (18.7-37.4)
INR (n=10)	2.2 (1.79-3.71)
Glycemia (mg/dL) (n=23)	111.0 (85.3-134)
Internal medium values	
Fraction of inspired oxygen (n=19)	40.0 (28.0-65.0)
Partial pressure of oxygen (mmHg) (n=18)	68.6 (58.2-90.7)
PaO_2_/FiO_2_ (n=16)	220.0 (110-286)
pH (n=19)	7.2 (7.09-7.34)
HCO_3_ (meq/L) (n=19)	12.2 (8.20-19.6)
pCO_2_ (mmHg) (n=19)	26.9 (23.6-34.1)
Lactate (mmol/L) (n=13)	11.1 (2.30‒16.0)

IQR: interquartile range, AST: aspartate transaminases, ALT: alanine transaminases, INR: international normalized ratio.

### Management of dengue cases

Patient management at the onset of signs and symptoms included mainly crystalloid fluids, sodium chloride solution 9°/00 (91.7%); the use of vasoactive drugs and mechanical ventilation was frequent (70.8%), analgesics (58.3%) and doses <300mg/24h of intravenous corticosteroids (45.8%) were also reported. Transfusion of blood derivatives or more than one blood product at the same time was reported in 46% of patients (Table 4).

**Table 4:** Treatment and management of cases of death due to dengue.

Treatment	n ^a^ (%)
Fluid management and active therapy	
Fluid therapy with crystalloids	22 (91.7)
Vasoactive drugs	17 (70.8)
Mechanical ventilation	18 (70.8)
Sedation and analgesia	14(58.3)
Intravenous corticosteroids	11(45.8)
Transfusion	
Red blood cell transfusion	11 (45.8)
Platelet transfusion	4 (16.8)
Fresh frozen plasma transfusion	6 (25.0)
Cryoprecipitate transfusion	4 (16.8)
Antipyretics	
Paracetamol	7 (29.2)
Metamizole	4 (16.8)
Paracetamol and metamizole	1 (4.2)
No antipyretics	5 (20.8)
Antibiotics	
3rd generation cephalosporin	2 (8.0)
Quinolones	2 (8.0)
Combination of two antibiotics	10 (41.6)
More than two antibiotics	7 (29.1)
No antibiotics	3 (12.5)

a non-excluding frequencies

The use of analgesics such as paracetamol was reported in 25% and metamizole in 17% of patients who died of dengue. Physical means were not used in any case. Antibiotics were widely used, 71% of patients received more than two classes of antibiotics (Table 4). Regarding transfusion, 45.8% of the patients received blood and 25% fresh frozen plasma. Platelet units and cryoprecipitate were also transfused in 16.8% of cases.

### Aspects related to the causes of death

PaO_2_/FiO_2_ values of 220 indicate pulmonary involvement; the pH was 7.22 which suggests severe acidosis. High lactate levels were reported with a median of 11.1 mmol/L (Table 3). Finally, 100% of the deaths presented neurological, respiratory and cardiovascular disorders. Hematologic disorders were reported in 58.3% and gastrointestinal and renal dysfunction in 50% of the cases.

## DISCUSSION

Of the 24 cases, most were women who had a history of *diabetes mellitus* and arterial hypertension. The time to admission in severe cases was long and the most frequent symptoms were fever, nausea and vomiting. The most frequent laboratory findings were leukocytosis, metabolic acidosis and hyperlactatemia. Treatment in a specialized unit was late and antibiotics were used empirically.

Our findings are consistent with the strong association of the female sex with dengue shock syndrome previously reported ^8^ and may be explained by gender differences in seeking medical care, as well as physical characteristics ^9^. Three cases of dengue were reported in pregnant women, which were not categorized as dengue cases at hospital admission. Therefore, a correct diagnosis is important, especially to avoid perinatal involvement with high morbidity in pregnant women, especially during the first two trimesters ^10^.

First contact with a healthcare facility was with a high complexity hospital in Piura in 58% of the cases; it is important to note that 16.7% of the cases began receiving treatment in a primary care center. Fatal cases of dengue vary between 1% and 3% in countries near to Peru^6^.

The highest mortality rate was found in the age group 19 to 35 years and in those older than 65 years, totaling 75%. On the other hand, greater incidence has been reported in children and young adults ^11^, which differs from our results. The time elapsed between the first contact to healthcare and death had a mean of 4.10 (SD: 5.34) days, which shows the late start of treatment in these cases.

Fever, vomiting and abdominal pain were the most frequent signs and symptoms in the deceased patients. Severe forms of dengue infection are characterized by hemorrhage, hypotension, thrombocytopenia and plasma leakage, and may be accompanied by some neurological alterations. These signs can lead to shock and subsequent multisystem failure, which may have increased mortality, especially if comorbidities are present ^12^. Temperature during the febrile phase is usually ≥38.5°C and our results showed an average temperature of ≥37.8°C, this difference could be explained by the fact that patients could be transitioning from the febrile phase to the critical phase ^13^^)^ or by the antipyretic effect.

The daily platelet count appears to be a promising predictor for dengue shock syndrome ^14^, although only half of the patients showed this finding. Serum lactate was high in this sample.

Intensive monitoring and good supportive care can reduce mortality rates to below 1%. The sepsis survival campaign is based on the sepsis and septic shock survival guidelines ^15^; this program has not been validated and often cannot be applied to low- and middle-income countries, where the frequency of sepsis is high and sepsis outcomes are often poor ^16^. Cases of severe dengue require treatment with fluids, according to the MINSA technical guideline^7^^)(^^16^. Therapy with blood products is indicated in patients with major bleeding, but there is controversy regarding platelet transfusions for thrombocytopenia without bleeding ^17^.

On the other hand, previous antibiotic treatment and empirical use of antipyretics are common in patients with clinical symptoms compatible with dengue, which could delay the diagnosis, care or management of dengue cases. On the other hand, although guidelines suggest avoiding the use of metamizole ^18^, we found that 17% of the cases used this analgesic. Likewise, the use of paracetamol as an antipyretic was reported in only 21% of cases. It should be noted that, although prophylactic antibiotic treatment is not recommended without evidence of bacterial infections ^19^, most patients (87.5%) had received some dose of antibiotics.

The mortality rate of dengue cases with untreated shock is 20%, this can decrease between 1% and 2.5% with adequate treatment ^20^, while, in Peru during 2017 it exceeded 35%. In this sample, 100% of the cases had shock with multiorgan failure. Shock is a medical emergency, and resuscitation with fluids as well as hemodynamic assessments are recommended within the first three hours ^15^.

A limitation of this study was that some medical records were missing and others had incomplete data. 

In conclusion, mortality from severe dengue fever was higher in adult women with classic symptomatology; likewise, the time of admission to the first healthcare facility to a specialized center was long.
