# Supplementary material for: Clinical-epidemiological characteristics of deaths due to dengue during an outbreak in northern Peru
Source: Rev Peru Med Exp Salud Publica. 2023 Mar 31;40(1):67–72. doi: 10.17843/rpmesp.2023.401.12148 (PMC10953633; doi:10.17843/rpmesp.2023.401.12148)
Supplement: Translation

# Translation: Características clínicas y epidemiológicas de las muertes por dengue durante un brote en el norte del Perú

**Authors:** Néstor Luque, Catia Cilloniz, María J. Pons, Fernando Donaires, Roger Albornoz, Moisés Mendocilla-Risco, Manuel Espinoza

**Affiliations:** 1 Escuela de Medicina Humana, Facultad de Ciencias de la Salud, Universidad Peruana Unión (UPeU), Lima, Perú.; 2 Universidad Continental, Lima, Perú.; 3 Grupo enfermedades infecciosas re-emergentes, Universidad Científica del Sur, Lima, Perú.; 4 Instituto Nacional de Salud, Lima, Perú.; 5 Universidad Nacional Mayor San Marcos, Lima, Perú.

**Keywords:** Dengue, Mortalidad, Registros Médicos, Epidemiología, Perú

## Abstract

Con el objetivo de describir las características clínicas y epidemiológicas de los pacientes fallecidos por dengue durante el 2017 en Piura, se realizó un estudio retrospectivo de revisión de 24 historias clínicas. El 67% de los casos fueron mujeres y tres (12,5%) estaban embarazadas. La diabetes (12,5%) y la hipertensión (16,7%) fueron las comorbilidades más frecuentes. Sólo en el 12,5% se reportó dengue previo. El tiempo transcurrido desde asistencia sanitaria hasta la muerte fue de 4,10 (DE: 5,34) días. Se hicieron transfusiones de glóbulos rojos en el 45,8% de los casos, plasma en el 25%, plaquetas en el 16,8% y crioprecipitado en el 16,8%. También fueron frecuentes la terapia con cristaloides (91,7%) y el tratamiento con fármacos vasoactivos (70,8%). En conclusión, la mortalidad del dengue grave fue mayoritaria en las mujeres adultas y el tiempo de atención desde el primer establecimiento de salud hasta una unidad especializada fue prolongada.

## INTRODUCCIÓN

En América, en el 2019, se registraron más de 2,7 millones de casos de dengue, de los cuales 22 127 fueron casos graves, con 1206 muertes ^1^. En la última década ha aumentado el número de muertes por dengue y varios países han registrado elevados índices de casos fatales durante las últimas epidemias ^2^. Desde su reingreso al Perú en 1990, el dengue ha sido un problema de salud creciente, reportándose la circulación de los cuatro serotipos del virus ^3^. Los reportes epidemiológicos mostraron que en el Perú hubo 68 290 casos confirmados y probables, de los cuales 27 249 (35,8%) fueron confirmados en laboratorio, 79 de los casos confirmados fallecieron, de estos 43 (54,4%) procedían de la región de Piura durante el 2017 ^4^. 

En el Perú, el fenómeno de El Niño Costero del 2017 fue bastante destructivo, especialmente para la región norte donde duró casi tres meses ^5^. Según la Organización Mundial de la Salud es necesario conocer los datos epidemiológicos mundiales para poder predecir e identificar los casos graves de dengue ^6^. Por este motivo, el objetivo del estudio fue describir las características clínicas y epidemiológicas de los pacientes fallecidos a causa del dengue en los establecimientos de salud de la región de Piura, en el norte del Perú en el 2017.

**MENSAJES CLAVE**

Motivación para realizar el estudio. Describir las características de los pacientes fallecidos por dengue grave durante el fenómeno de El Niño del 2017 en Piura.

Principales hallazgos. La mortalidad del dengue grave fue mayoritaria en las mujeres adultas, la primera atención se realizó en hospitales de mayor nivel y la atención para casos graves de dengue en una unidad especializada fue tardía.

Implicancias. La mortalidad del dengue grave fue mayoritaria en las mujeres adultas, la primera atención se realizó en hospitales de mayor nivel y la atención para casos graves de dengue en una unidad especializada fue tardía.

## EL ESTUDIO

### Población y diseño del estudio

Se realizó un estudio retrospectivo de revisión de las historias clínicas de los pacientes fallecidos por dengue en la región de Piura hasta la semana epidemiológica 42 del 2017, que incluye un brote de dengue. El Área de Epidemiología de la Dirección Regional de Salud de Piura (DIRESA-Piura), proporciono 32 historias clínicas, de los que se excluyeron ocho (historias repetidas, sin acta de defunción médica, información ilegible e información incompleta), por lo que finalmente se revisaron 24.

### Definición de caso de dengue

El diagnóstico de dengue se realizó con los criterios clínicos y epidemiológicos establecidos por la guía técnica del Ministerio de Salud (MINSA) ^7^. Todos los casos tuvieron resultados positivos en una o más de las pruebas establecidas para el diagnóstico, ya sea el ensayo inmunoenzimático (ELISA) para la glicoproteína no estructural NS1, o la detección de anticuerpos IgM o IgG con el kit peruano TARIKI-DENGUE y Standard Diagnostics BIOLINE Dengue Duo, respectivamente.

### Recolección de datos

Todos los casos pertenecen a la región de Piura. Los médicos encargados completaron los formularios del MINSA y el registro. La información de la historia clínica se obtuvo con una herramienta de recojo de datos, donde se incluyeron datos epidemiológicos como la edad, sexo, antecedentes patológicos, antecedentes de dengue, tiempo de enfermedad, lugar de atención y características del tiempo de atención entre establecimientos de salud; síntomas y signos como la fiebre (temperatura axilar mayor a 38° C), cefalea, dolor retroorbital, mialgia, artralgia, malestar general, otros; funciones vitales; manejo de fluidos; soporte vital y hematológico; exámenes de laboratorio (se usaron equipos automatizados para valorar gasometría); uso de drogas antitérmicas y antibióticos.

### Análisis estadístico

Las variables continuas se reportaron con la media y desviación estándar (DE) o con la mediana y el rango intercuartílico (RIC), dependiendo de su distribución normal. Las variables categóricas se reportaron con frecuencias y porcentajes. Todos los análisis estadísticos se realizaron con el programa estadístico SPSS 21.

### Aspectos éticos

El estudio tiene la aprobación del Comité de Ética de Investigación de la Universidad Peruana Unión (UPeU) (código: 2022-CEUPeU-001). 

## HALLAZGOS

### Diagnóstico y atención de casos de dengue

De los 24 casos, las pruebas positivas para NS1 (ELISA), IgM (ELISA) e IgG (ELISA) se dieron en el 62,5%, 70,8% y 41,7%, respectivamente. Según los registros médicos, 8 (33,3%) muertes ocurrieron en el Hospital Santa Rosa II-2 del MINSA, 6 (25,0%) en el Hospital Cayetano Heredia III de EsSalud, 5 (20,8%) en otros hospitales de EsSalud, 1 (4,2%) en la sanidad de las Fuerzas Armadas y 4 (16,7%) ocurrieron en un centro de atención primaria. 

### Características clínicas y epidemiológicas de las muertes por dengue

Las características epidemiológicas de los pacientes que murieron por dengue se describen en la Tabla 1. La mortalidad en los pacientes de sexo femenino fue mayor; del total, 3 (12,5%) fallecidas estaban embarazadas. Comorbilidades como diabetes *mellitus* e hipertensión arterial fueron reportadas en 12,5% y 16,7% respectivamente, no se encontró el uso de corticoides previos al ingreso. Como posible contacto previo con un paciente con dengue se identificaron a 4 (16,7%) (1 caso con síndrome febril y 3 casos con historia previa de dengue). Según los diagnósticos clínicos al ingreso, 6 (25%) tuvieron dengue con signos de alarma y en 10 (42%) tuvieron dengue severo. La media del tiempo que transcurrió entre la primera atención en el establecimiento de salud y el fallecimiento fue de 4,10 (DE 5,34) días (Tabla 1).

**Tabla 1:** Características epidemiológicas de los casos de muerte por dengue.

Variables	n (%)
Edad (años) ^a^	46 (25,0) (13-88) ^b^
Sexo	
Femenino ^c^	16 (67,0)
Masculino	8 (33,0)
Comorbilidades	
Diabetes *mellitus*	3 (12,5)
Hipertensión arterial	4(16,7)
Otros	6(25,0)
No consigna	11(45,8)
Historia previa de dengue	
Sí	3 (12,5)
Síndrome febril	1 (4,2)
No información	20 (83,3)
Diagnostico en admisión (primer ES)	
Dengue con síntomas de alarma	6 (25,0)
Dengue severo	10 (41,6)
Síndrome febril	4 (16,7)
Otro diagnóstico ^d^	4 (16,7)
Tiempo de atención (días)	
Desde el ES al fallecimiento ^a^	4,10 (5,34)
Desde el inicio de la enfermedad al fallecimiento ^a^	7,93 (5,97)

a Media y desviación estándar, ^b^ mínimo‒máximo ^c^ tres mujeres fueron gestantes, ^d^ neumonía comunitaria grave (2 casos) y gestantes con preeclampsia severa (2 casos).

ES: establecimiento de salud.

Las características clínicas al ingreso al establecimiento de salud se describen en la Tabla 2. Las manifestaciones clínicas más frecuentes se relacionaron con la fase febril del dengue: fiebre (87,5%) y síntomas sistémicos como cefalea (33,3%), náuseas/vómitos (58,3%), dolor abdominal (45,8%), artralgia (33,3%) entre otros. Además, los signos vitales en el momento del ingreso revelan taquicardia (frecuencia cardíaca media de 108 latidos por min), polipnea (frecuencia respiratoria media de 25.

**Tabla 2:** Características clínicas en el momento del ingreso relacionadas con las muertes por dengue.

Variables	n (%)
Síntomas y signos	
Fiebre	21 (87,5)
Cefalea	8 (33,3)
Dolor retroorbital	3 (12,5)
Mialgia	1 (4,2)
Artralgia	8 (33,3)
Malestar general	9 (37,5)
Dolor abdominal	11 (45,8)
Erupción	1 (4,2)
Nausea/vómito	14 (58,3)
Petequias	2 (8,3)
Ictericia	2 (8,3)
Hematemesis	1 (4,2)
Diarrea	5 (20,8)
Convulsiones	3 (12,5)
Insuficiencia respiratoria	2 (8,3)
Melena	2 (8,3)
Alteraciones de la conciencia	3 (12,5)
Otros ^a^	4 (16,7)
Signos vitales (en admisión)	
Temperatura corporal (°C) ^b^	37,8 (0,9)
Rítmo cardíaco (pulso/min) ^b^	108 (19,5)
Presión arterial media (mmHg) ^b^	75 (21,7)
Frecuencia respiratoria (min) ^b^	25 (10,3)
Escala de Glasgow ^b^	13 (2,9)

a Ascitis (un caso), palidez (un caso), sudoración profusa (un caso) y derrame pleural (un caso), ^b^ media y desviación estándar.

Los resultados del laboratorio se describen en la Tabla 3. El hemograma se realizó dentro de las 48 horas del ingreso en la asistencia sanitaria, reportando una amplia variabilidad, debido a que los datos tomados de establecimientos de salud periféricos fueron hechos en forma manual y los de mayor complejidad se realizaron con métodos sistematizados. Los resultados han mostrado una tendencia a disminuir los rangos de conteo de plaquetas. El lactato mostro niveles altos. Las enzimas transaminasas AST y ALT fueron elevadas, lo que indica alteraciones hepáticas. El INR fue elevado. La mitad de los pacientes presentaron trombocitopenia.

**Tabla 3:** Resultados de los parámetros de laboratorio de los casos de muerte por dengue.

Variables	Mediana (RIC)
Parámetros hematológicos	
Leucocitos/mm^3^ (n=24)	6620 (4142-11692)
Hematocrito (%) (n=24)	41,0 (29,7-45,5)
Hemoglobina (gr/dL) (n=24)	13,6 (10,3-15,3)
Creatinina (mg/dL) (n=21)	1,3 (0,9-2,2)
Urea (mg/dL) (n=22)	53,5 (29,5-71,5)
Sodio sérico (meq/L) (n=19)	140,3 (132-142)
Potasio sérico (meq/L) (n=19)	4,2 (3,85-5,45)
Bilirrubina total (mg/dL) (n=18)	0,7 (0,26-3,59)
Bilirrubina directa (mg/dL) (n=18)	0,4 (0,16-2,48)
Bilirrubina indirecta (mg/dL) (n=18)	0,3 (0,12-0,79)
Plaquetas/mm^3^ (n=23)	105 000 (47 000-136 500)
AST (mg/dL) (n=18)	504,0 (87,2-1694)
ALT (mg/dL) (n=18)	432,0 (82,2-1343)
Tiempo de protrombina (seg) (n=11)	21,5 (18,7-37,4)
INR (n=10)	2,2 (1,79-3,71)
Glicemia (mg/dL) (n=23)	111,0 (85,3-134)
Valores de medio interno	
Fracción inspirada en el oxígeno (n=19)	40,0 (28,0-65,0)
Presión parcial de oxígeno (mmHg) (n=18)	68,6 (58,2-90,7)
PaO_2_/FiO_2_ (n=16)	220,0 (110-286)
pH (n=19)	7,2 (7,09-7,34)
HCO_3_ (meq/L) (n=19)	12,2 (8,20-19,6)
pCO_2_ (mmHg) (n=19)	26,9 (23,6-34,1)
Lactato (mmol/L) (n=13)	11,1 (2,30‒16,0)

RIC: rango intercuartilico, AST: Aspartato transaminasas, ALT: Alanina transaminasas, INR: relación internacional normalizada.

### Manejo de los casos de dengue

El manejo de los pacientes al inicio de los signos y síntomas fue principalmente con líquidos cristaloides; solución de cloruro de sodio 9 º/00 (91,7%), el uso de drogas vasoactivas y ventilación mecánica fue frecuente (70,8%), también se reportaron analgésicos (58,3%) y dosis <300mg/24h de corticoides intravenosos (45,8%). En el 46% de los pacientes se reportó la transfusión de derivados sanguíneos o más de un elemento de la sangre al mismo tiempo (Tabla 4).

**Tabla 4:** Tratamiento y manejo de los casos de muerte por dengue.

Tratamiento	n ^a^ (%)
Manejo de fluidos y terapia activa	
Terapia de fluidos con cristaloides	22 (91,7)
Drogas vasoactivas	17 (70,8)
Ventilación mecánica	18 (70,8)
Sedación y analgesia	14(58,3)
Corticoesteroides endovenosos	11(45,8)
Transfusión	
Transfusión de glóbulos rojos	11 (45,8)
Transfusión de plaquetas	4 (16,8)
Transfusión de plasma fresco congelado	6 (25,0)
Transfusión de crioprecipitado	4 (16,8)
Antipiréticos	
Paracetamol	7 (29,2)
Metamizol	4 (16,8)
Paracetamol y metamizol	1 (4,2)
No antipiréticos	5 (20,8)
Antibióticos	
Cefalosporina de 3ª generación	2 (8,0)
Quinolonas	2 (8,0)
Combinación de dos antibióticos	10 (41,6)
Más de dos antibióticos	7 (29,1)
No antibióticos	3 (12,5)

a Frecuencias no excluyentes

El uso de analgésicos como el paracetamol se reportó en el 25% y el metamizol en el 17% de los pacientes que murieron de dengue. No se encontró la aplicación de medios físicos. Se describe un alto uso de antibióticos, ya que el 71% de los pacientes recibieron más de dos familias de antibióticos (Tabla 4). Para la transfusión, el 45,8% recibió sangre y el 25% plasma fresco congelado. También se transfundieron unidades de plaquetas y crioprecipitado en el 16,8% de los casos.

### Aspectos relacionados con las causas de muerte

Los valores de PaO_2_/FiO_2_ de 220, indican compromiso pulmonar, el medio interno mostro un pH de 7,22 lo que sugiere acidosis severa. Se reportó niveles altos de lactato con una mediana de 11,1 mmol/L (Tabla 3). Finalmente, el 100% de las muertes presentaron disfunciones neurológicas, respiratorias y cardiovasculares. El 58,3% presentaron disfunción hematológica y el 50% disfunción gastrointestinal y renal.

## DISCUSIÓN

De los 24 casos; la mayoría fueron mujeres, tuvieron antecedentes de diabetes *mellitus* e hipertensión arterial, los tiempos de atención en las formas graves fueron tardías, los síntomas más frecuentes fueron fiebre, náuseas y vómitos. Los resultados de laboratorio más frecuentes fueron leucocitosis, acidosis metabólica e hiperlactademia. El abordaje en una unidad especializada fue tardío y se usó antibióticos empíricamente.

Se reporta predominio de mortalidad en el sexo femenino, esto concuerda con la fuerte asociación del sexo femenino con el síndrome de choque del dengue previamente reportado ^8^ y puede ser explicado por las diferencias de género en la búsqueda de atención médica, así como las características físicas ^9^. Se han reportado tres casos de dengue en mujeres embarazadas, que no fueron catalogados como casos de dengue al inicio de la atención hospitalaria. Por lo tanto, es importante un diagnóstico correcto especialmente para evitar la afectación perinatal con alta morbilidad en la mujer embarazada sobre todo en los dos primeros trimestres ^10^. 

Los resultados mostraron que en el 58% de los casos, su primera aproximación a un establecimiento de salud fue a los hospitales de mayor complejidad de Piura, pero se destaca que el 16,7% de los casos tuvo la primera atención en un centro de atención primaria. Los casos fatales de dengue varían entre 1 a 3% en países cercanos al Perú ^6^.

La mayor tasa de mortalidad se produjo en el grupo de edad de 19 y 35 años y en mayores de 65 años, sumando 75%. Contrariamente, se atribuye una mayor afectación en niños y adultos jóvenes ^11^, lo que no ha ocurrido en el presente estudio. El tiempo transcurrido entre la primera atención y la muerte de los pacientes tuvo una media de 4,10 (DE: 5,34) días, lo que se representa un abordaje tardío en el manejo de los casos.

En cuanto a los signos clínicos, la fiebre, los vómitos y el dolor abdominal fueron los signos y síntomas más frecuentes en los pacientes fallecidos. Las formas graves de infección por dengue se caracterizan por la presencia de hemorragias, hipotensión, trombocitopenia y fuga de plasma, y pueden acompañarse de algunas alteraciones neurológicas. Estos signos pueden producir un choque y posterior fallo multisistémico, que podría tener una mayor mortalidad, especialmente si hay comorbilidades presentes ^12^. Se reportó en promedio una temperatura ≥37,8ºC, según lo descrito por la teoría la fase febril es generalmente ≥38,5ºC, esta diferencia se explicaría por el hecho de que los pacientes podrían estar pasando de la fase febril a la fase crítica ^13^ o por el efecto antipirético.

El conteo diario de plaquetas parece ser prometedor para predecir el síndrome de choque por dengue ^14^, aunque sólo se observó en la mitad de los pacientes cuando fueron atendidos en el hospital. El lactato sérico en esta muestra fue alto.

Está demostrado que un monitoreo intensivo y una buena atención de apoyo pueden reducir las tasas de mortalidad por debajo del 1%. La campaña de supervivencia en sepsis se basa en las pautas de supervivencia en la sepsis y en el shock séptico ^15^, este programa no ha sido validado y a menudo no es posible usarlo en la práctica en los países de ingresos bajos y medios, donde la frecuencia de sepsis es alta y los resultados de esta son a menudo pobres ^16^. Los casos de dengue severo requieren tratamiento con fluidos, de acuerdo con el estándar técnico del MINSA (7) ^16^. La terapia con componentes sanguíneos está indicada en pacientes con hemorragia importante, pero existe una controversia con las transfusiones de plaquetas para la trombocitopenia sin sangrado ^17^. 

Por otro lado, la medicación previa con antibióticos y el uso empírico de antipiréticos son comunes en pacientes con síntomas clínicos compatibles con el dengue, lo que podría retrasar el diagnóstico, la atención o el manejo de los casos de dengue. Por otra parte, aunque las guías sugieren evitar el uso de metamizol ^18^, encontramos el uso de este analgésico en el 17%. Por otra parte, el uso de paracetamol como antipirético fue reportado en sólo el 21% de los casos. Se debe destacar que, aunque no se recomienda el tratamiento antibiótico profiláctico sin evidencia de infecciones bacterianas ^19^, la mayoría de los pacientes (87,5%) había recibido alguna dosis de antibióticos. 

La tasa de mortalidad de dengue con shock no tratado es de 20%, esto se reduce entre el 1% y el 2,5% con un tratamiento adecuado ^20^, mientras que, en el Perú durante el año 2017 superó el 35%. En esta muestra el 100% presento shock con fallo multiorgánico. El shock es una emergencia médica, está recomendado que antes de tres horas se debe hacer la resucitación con fluidos y mediciones hemodinámicas ^15^.

Como una limitación de este estudio se debe reconocer la ausencia de algunas historias clínicas o la falta de datos completos de algunos casos. 

En conclusión, la mortalidad del dengue grave fue a predominio de mujeres adultas, y con sintomatología clásica, asimismo, el tiempo de atención desde el primer establecimiento de salud hasta una unidad especializada fue prolongada.
